# CHST4 might promote the malignancy of cholangiocarcinoma

**DOI:** 10.1371/journal.pone.0265069

**Published:** 2022-03-16

**Authors:** Guanran Zhang, Xuyue Liu, Aiwen Jian, Kexin Zheng, Haiyan Wang, Jing Hao, Sujuan Zhi, Xiaoli Zhang

**Affiliations:** 1 Key Laboratory for Experimental Teratology of Ministry of Education, Department of Histology & Embryology, School of Basic Medical Sciences, Shandong University, Jinan, Shandong, China; 2 Department of Radiology, Shandong Provincial Hospital Affiliated to Shandong First Medical University, Jinan, Shandong, China; Texas A&M University, UNITED STATES

## Abstract

**Background:**

Cholangiocarcinoma (CCA) is reported as an aggressive cancer which leads to high mortality and no effective therapeutic target has yet been discovered. Surgical resection is the main method to treat patients with CCA. However, only one-third of CCA patients have the opportunity to accept the operation, leading to poor prognosis for CCA patients. Therefore, it is necessary to search for new therapeutic targets of CCA or core genes involved in the happening and growth of CCA.

**Aim:**

In this study, we utilized bioinformatics technology and accessed to several medical databases trying to find the core genes of CCA for the purpose of intervening CCA through figuring out an effective curative target.

**Methods:**

Firstly, three differentially expressed genes (DEGs) were discovered from GEPIA, and by further observing the distribution and gene expression, CHST4 was obtained as the core gene. Afterwards, correlated genes of CHST4 in CCA were identified using UALCAN to construct a gene expression profile. We obtained PPI network by Search Tool for the Retrieval of Interacting Networks Genes (STRING) and screened core genes using cytoscape software. Functional enrichment analyses were carried out and the expression of CHST in human tissues and tumors was observed. Finally, a CCA model was established for qPCR and staining validation.

**Results:**

Three differentially expressed genes (DEGs), CHST4, MBOAT4 and RP11-525K10.3, were obtained. All were more over-expressed in CCA samples than the normal, among which the change multiple and the gene expression difference of CHST4 was the most obvious. Therefore, CHST4 was selected as the core gene. We can see in our established protein–protein interaction (PPI) network that CHST4 had the highest degree of connectivity, demonstrating its close association with CCA. We found that genes were mainly enriched in CCs in the PPI networks genes which shows functional enrichment analysis results, including golgi lumen, extracellular space and extracellular region. CHST4 was found very specifically expressed in the bile duct and was significantly different from that in normal tissues. The overexpression of CHST4 was further verified in the established animal model of TAA-induced CCA in rats. Quantitative PCR (qPCR) demonstrated that CHST4 was significantly overexpressed in tumor tissues, verifying the role of CHST4 as the core gene of CCA.

**Conclusion:**

CHST4 was increasingly expressed in CCA and CHST4 is worth being studied much further in the intervention of CCA.

## Introduction

Cholangiocarcinoma (CCA) is a malignant tumor originating from the biliary ducts [1, 2]. It can be divided into intrahepatic cholangiocarcinoma (iCCA) and extrahepatic cholangiocarcinoma (eCCA) according to its anatomical location [3]. CCA accounts for about 3%-5% of all malignant tumors in the gastrointestinal system and the incidence of CCA is on the rise [4, 5].

There are many risk factors for CCA, such as bile duct cysts, chronic HBV and HCV infections, primary sclerosing cholangitis and liver cirrhosis [6, 7]. CCA which is a militant tumor leading to poor prognosis is one of the deadliest cancers [8]. The efficacy of chemotherapy is not good, and its survival time is less than one year on average. Surgical resection is the primary pathway of potential cure for CCA [9]. However, most advanced patients cannot be surgically treated and only about one-third of CCA patients are eligible for surgery [10]. The main factors leading to poor prognosis of CCA are advanced diagnosis and confined treatment options [11]. Therefore, there is still an urgent need to develop innovated treatments for CCA.

To synthesize a functional gene product, the process of gene expression is necessary to use the information from a gene. Through the investigation of the differential expression of genes in the tumor tissue and normal tissue, it is possible to find the most important genes in the growth and advancement of cancer. In current study, differentially expressed genes (DEGs) have been utilized to discover novel prognostic biomarkers in a variaty of cancers. 36 patients with CCA were involved in a recent study and their gene expression profiles and clinical features were analyzed by Junyu Long, et al. in 2021, to identify the DEGs of CCA. In this research, 78 hub genes associated with the differentiation of tumor were identified, among which 17 ones were considered prognostic biomarkers for CCA patients [12]. It is widely acknowledged that DEGs of CCA could be regarded as potential molecular therapy target for CCA. CHST4 is expressed in the pancreas, fallopian tube, gallbladder and liver according to the Human Protein Atlas [13]. CHST4 is one of the family members of GlcNAc6ST family genes. The transfer of sulfate can be catalyzed to GlcNAc residues position 6 which is non-reducing by CHST4. GlcNAc residues position 6 can function as L-selectin ligands in mucin-associated glycans. In high endothelial cells (HEVs), these L-selectin ligands are found crowded, leading to the orienting of lymphocyte [14].

Previous studies have identified the role of this gene in hepatocellular carcinoma. In addition, mounting evidence indicated that the expression level of CHST4 is tightly connected with the differentiation, development and progression of various malignant cancers. Study conducted by Longshan Zhang, et al. implies that CHST4 may recruit immune cells into tumor microenvironment, which prevent hepatitis B virus-related hepatocellular carcinoma (HBV-HCC) tumors from progressing. Immune cells include dendritic cells, CD4+ T cells, neutrophils, and macrophages [15]. In another study about the gene expression profiles of human intrahepatic cholangiocarcinoma by Natini Jinawath, et al., an abnormal increment of the expression level of CHST4 was revealed, confirming the important role CHST4 plays in CCA [16].

However, the link between the gene and CCA has not been explored [17], and the precise function and prognostic value of CHST4 in CCA remain unclear. In this study, effective curative targets and prognostic signals for who suffered from CCA were identified. CHST4 was regarded as a potential therapeutic gene and follow-up experiments were carried out.

## Materials and methods

### Processing of differentially expressed genes

Zhang’s Lab of Peking University (http://gepia.cancer-pku.cn/index.html) invented The Gene Expression Profiling Interactive Analysis (GEPIA). It is an online tool including 9,736 cancers and 8,587 normal samples, where the RNA sequencing expression data can be analyzed. The differentially expressed genes (DEGs) can be figured out using GEPIA. In our study, we use this method to define the DEGs from normal and CCA samples. We filtered out the DEGs according to the percentage cutoff and |log_2_FC|. Genes with |log_2_FC| ≥ 3.0 and percentage cutoff = 0.9 were defined as DEGs. Then we observed gene expression profile and distribution of DEGs.

### Correlated genes of CHST4

UALCAN is an extensive and synergistic net materials, which was established by PERL-CGI using CSS and javascript with high quality graphics, which allows researchers to collect useful information and data about the interested genes. We used UALCAN tool to find correlated genes with CHST4 in CCA. Pearson correlation coefficient was calculated and the six genes most associated with CHST4 were screened. Gene expression in CCA based on sample types was obtained from UALCAN.

### PPI network establishment and central genes identification

The Search Tool for the Retrieval of Interacting Networks Genes (STRING) database (http://string-db.org/) was employed to build our protein–protein interaction (PPI) networks. We regarded 0.4 as the minimum which limited PPI pairs as the lowest interaction score. In order to dig out possible relationship of PPI, we entered CHST4, which was obtained before, into the STRING database. Moreover, we utilized cytoscape software to adjust and revamp the PPI network (www.cytoscape.org/). We also calculated different connectivity degree. If connectivity degrees were important between each node, the protein nodes got a high mark. The plugin cytoHubba of cytoscape software is used to calculate the marks. Thus, we identified the top 3 genes whose connectivity degree rank the highest as central genes.

### GO and KEGG pathway analysis

Biological process (BP), cell component (CC) and molecular function (MF), usually regarded as the results of functional enrichment studies in large dimensions, can show the function of gene bod, so we analyze them through Gene Ontology (GO) analysis. Kyoto Encyclopedia of Genes and Genomes (KEGG), as one of the most famous database resource of biological information, which is utilized in exploring not only biological systems but also advanced functions from information of molecular level, especially genomic sequencing gained from huge molecular datasets. We utilized database including biological tools and data to analyze and serving as an online bioinformatics database, which is Database for Annotation, Visualization and Integrated Discovery (DAVID) v6.8. It enables users to get the results of GO analysis and KEGG pathway enrichment analysis of the PPI networks genes by obtaining biological information and analyzing gene and protein functional annotation information(https://david.ncifcrf.gov/). WEB-based Gene SeT AnaLysis Toolkit (WebGestalt) was used for enrichment analysis in three well-constructed and completing modes, which is Over-Representation Analysis (ORA), Gene Set Enrichment Analysis (GSEA), and Network Topology-based Analysis (NTA). Also, we gain GO analysis and KEGG pathway enrichment analysis of the PPI networks genes using WebGestalt.

### Expression of CHST in human tissues and tumors

We used UALCAN online tool to compare the appearance of CHST4 in various tumors and normal samples. Swedish-based program invented The Human Protein Atlas (HPA) for the purpose to represent the whole proteins in human. The consolidation of different omics technologies was used in it, such as mass spectrometry-based proteomics, transcriptomics antibody-based imaging, and systems biology (https://www.proteinatlas.org). We got CHST4 expression data in cells and tissues from HPA.

### Experimental protocol

We used 108 male Sprague-Dawley (SD) rats (290-310g) in our study and divided all animals into two groups, which comprised of the experiment group (n = 54) and the control group (n = 54). We kept animals in the room, exposed to light for 12 hours (07:00–19:00), at surrounding temperature of 21 ± 1 degree Celsius, with plenty of food and water. In the experiment, we fed rats 300 mg thioacetamide (TAA)/L in drinking water until they were killed [17]. We monitored rats for signs of pain during the study, and when necessary, buprenorphine was used to lessen their distress. We harvested six animals (three experiment group rats and three control group rats) per week during this research to observe the effect of TAA and build the cholangiocarcinoma animal model. In this study, we weigh the rats weekly to see weight change.

### Collection and hematoxylin-eosin staining of the liver

We used isoflurane inhalation to deeply anesthetize the animals before the surgery. We put rats into the gas-tight anesthesia chamber in which rats were exposed to 2% (1~1.5 minimum alveolar concentration) isoflurane (in 4L/min O2) for 10 minutes. After that, the animals were inconscient, and if they gave little response to tail pinch, we removed them from the chamber. The rats were permitted to breathe indoor air on their own and we used heating pads to keep their rectum temperature at 37 degrees Celsius. After midline laparotomy, we explored and carefully examined all liver lobes to determine the presence of CCA. Midline laparotomy and hepatectomy results in the death of anesthetized rats with less distress. We fixed the liver samples in 10% formalin and parted liver embed in paraffin at 4 μm thickness. Then we used hematoxylin-eosin and masson to stain our samples for accurate observation. We captured images to observe CCA cells on Olympus VS-120 virtual digital slide scanning system with 40x objectives. We chose automatic scanning mode to automatically scan all slides (1–5 slides) on the stage. The multi-point intelligent focusing formed focused topographic maps solving the problem of the different flatness. The digital images were obtained for accurate observation and analysis.

### Extraction of RNA and real-time quantitative polymerase chain reaction

We obtained the whole RNA by trizol method and we calculated relative CHST expression by 2−^ΔCt^ (ΔCt = Ct of the CHST4 subtract Ct of glyceraldehyde-3-phosphate dehydrogenase). TriZol reagent (Invitrogen) was prepared for the whole RNA. We rigorously followed the manufacturer’s instructions. We used the SuperScript II Reverse Transcriptase Kit (Invitrogen) to reverse transcribe the CCA RNA samples and normal RNA samples into cDNA. The volume contents 10 pM of each primer. We added 1 μl reverse transcription product as the manufacturer’s instructions. And the total volume is 25 μl.

### Ethics approval

The methods of research were approved by the Ethics Committee of the School of Basic Medical Sciences of Shandong University. Operations have been performed according to the Declaration of Helsinki and the ARRIVE guidelines (ECSBMSSDU2021-2-82). The informed consent was not applicable.

## Results

### Identification of DEGs

**Fig 1:**
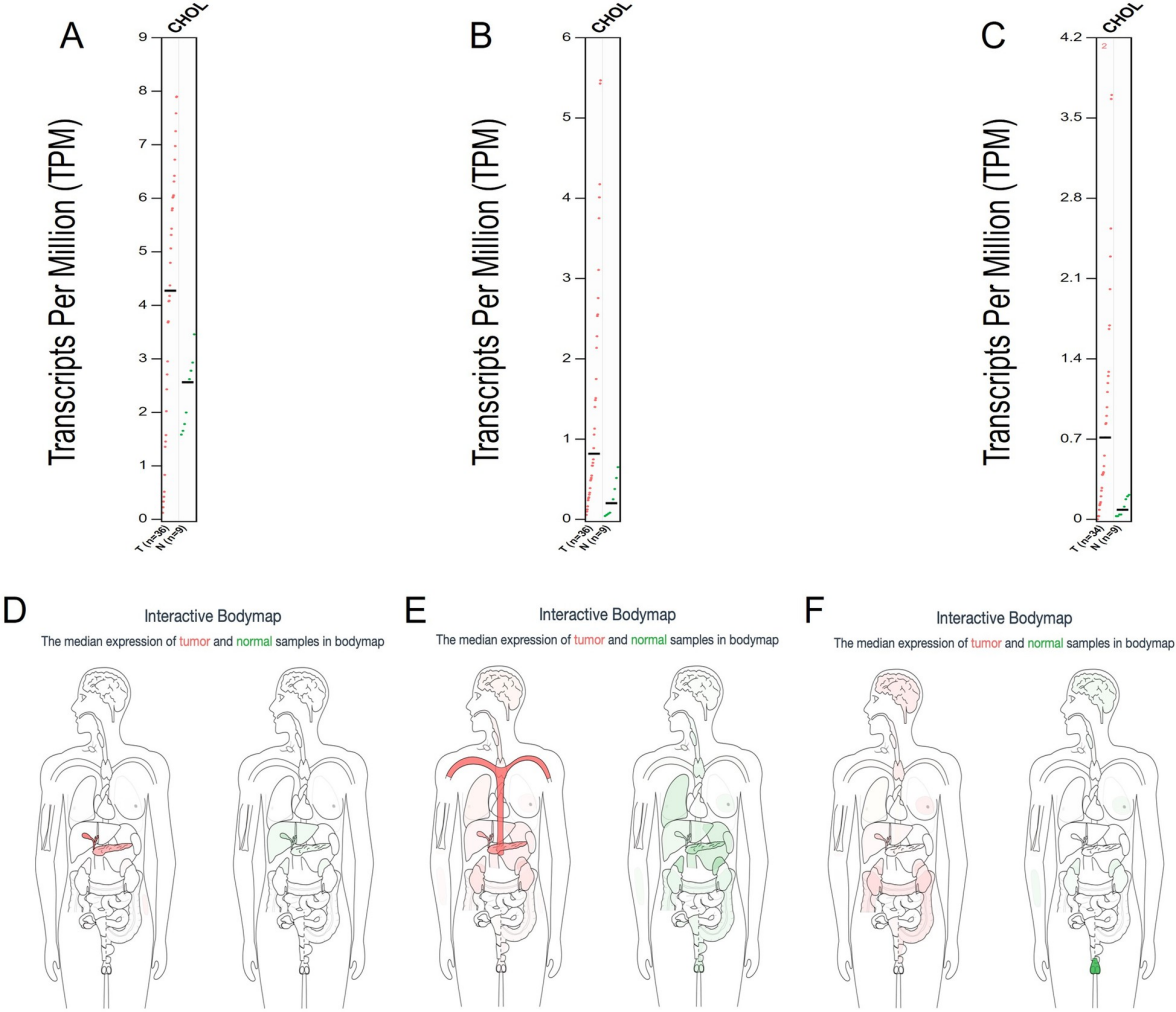
Gene expression profile and distribution of DEGs from the GEPIA. (A) CHST4 expression profile, (B) MBOAT4 expression profile, (C) RP11-525K10.3 expression profile, (D) distribution of CHST4, (E) distribution of MBOAT4, (F) distribution of RP11-525K10.3. The red means tumor samples and green means normal samples in the bodymap and expression profile.

Based on the percentage cutoff and |log_2_FC|, three differentially expressed genes (DEGs) were obtained (Table 1). Compared to normal tissues, all were over-expressed in tumor tissues. Among them, change multiple of CHST4 was the most obvious. Afterwards, gene expression profile and distribution of DEGs were acquired from the GEPIA (Fig 1). Based on gene expression profiles, the gene expression difference of CHST4 between tumor and normal samples was the most significant. According to the distribution of DEGs, CHST4 has the highest expression in the gallbladder, followed by the pancreas, while in other parts of the body the expression is relatively low, demonstrating extremely high specificity. The other two DEGs showed different distribution features. MBOAT4 is mainly expressed in the blood, with low expression in other parts of the body. RP11-525K10.3 is highly expressed in normal testis and obviously reduced in tumor tissue. Therefore, CHST4 was selected as the core gene for subsequent analyses.

**Table 1:** Information of the three DEGs from the GEPIA.

Gene Symbol	Gene ID	Median (Tumor)	Median (Normal)	Log2(Fold Change)	Percentage
CHST4	ENSG00000140835.9	236.336	22.351	3.345	1.000
MBOAT4	ENSG00000177669.3	42.071	3.92	3.130	1.000
RP11-525K10.3	ENSG00000259867.5	60.112	6.37	3.052	1.000

### Correlated genes of CHST4 and gene expression profile

**Fig 2:**
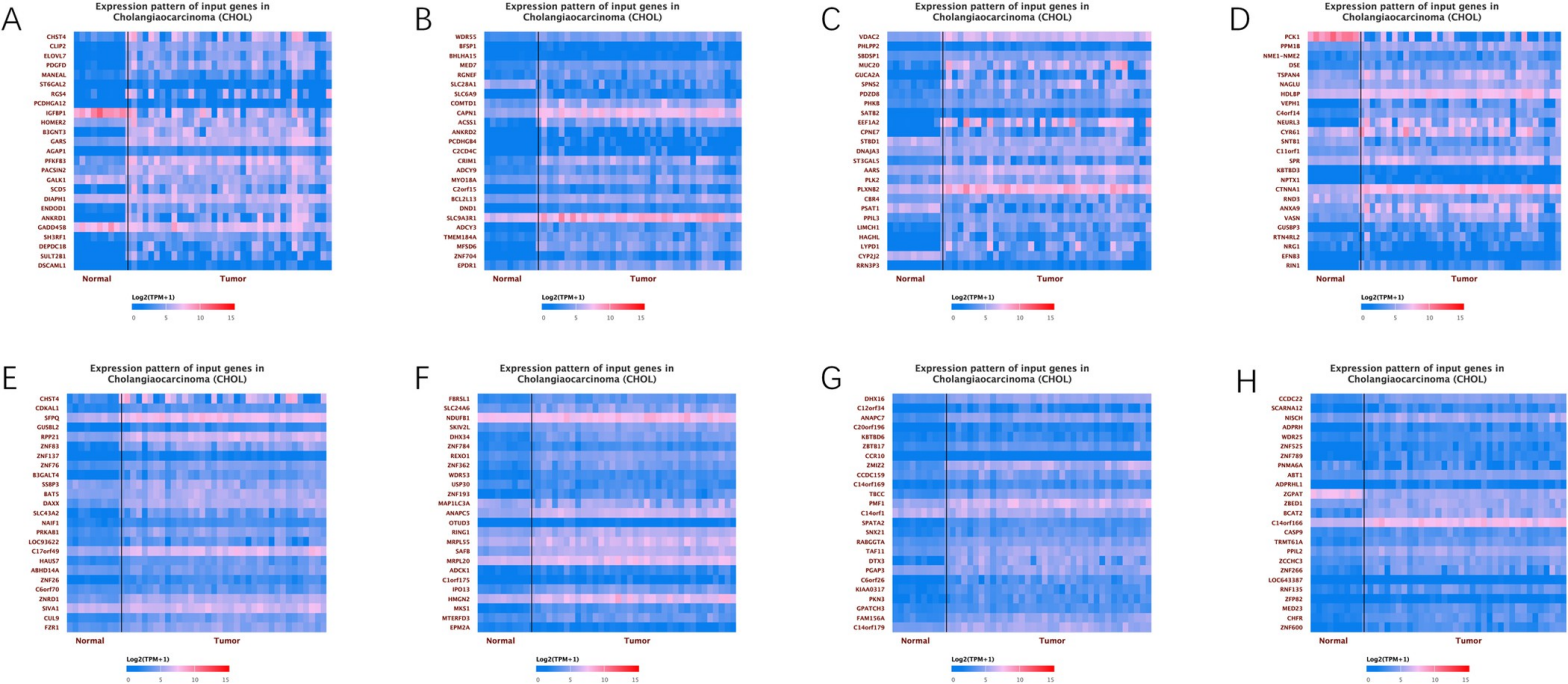
Expression pattern of correlated genes about CHST4 from the UALCAN. (A) top 1–25 genes positively correlated with CHST4 in CCA, (B) top 26–50 genes positively correlated with CHST4 in CCA, (C) top 51–75 genes positively correlated with CHST4 in CCA, (D) top 76–100 genes positively correlated with CHST4 in CCA, (E) top 1–25 genes negatively correlated with CHST4 in CCA, (F) top 26–50 genes negatively correlated with CHST4 in CCA. (G) top 51–75 genes negatively correlated with CHST4 in CCA. (H) top 76–100 genes negatively correlated with CHST4 in CCA. The red means high expression of genes and blue means low expression of genes in the heatmap.

**Fig 3:**
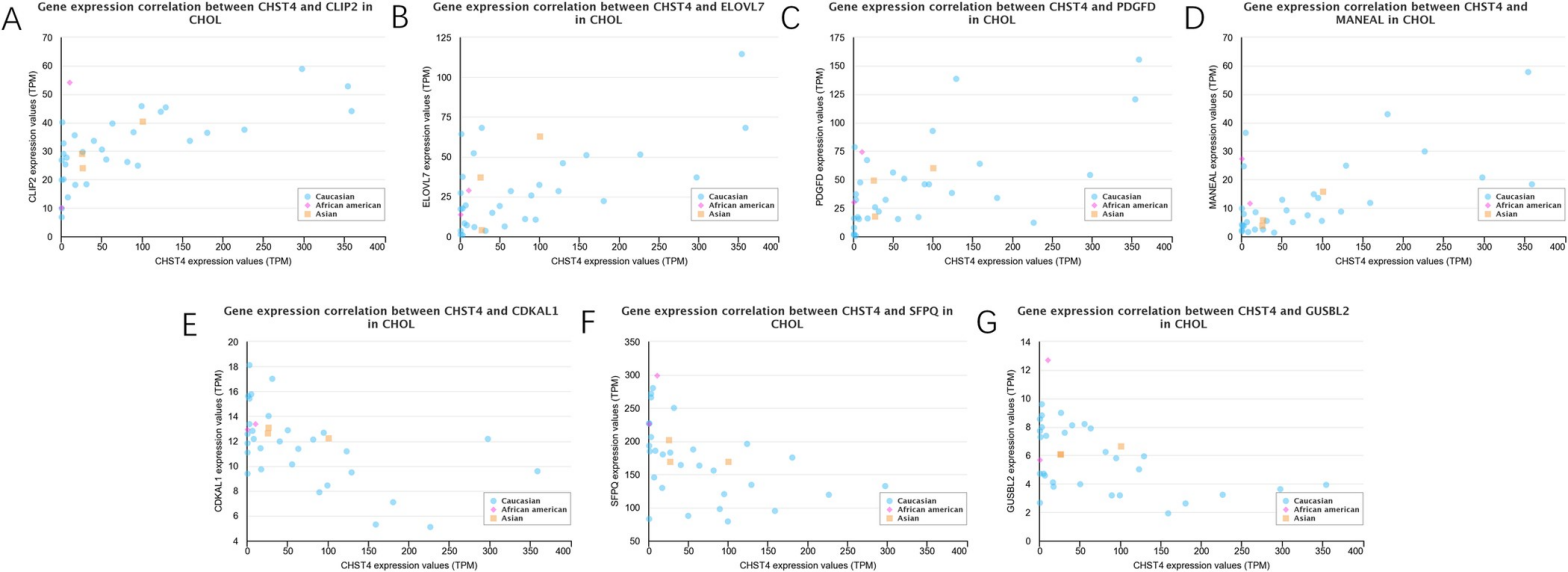
Scatter plot of gene expression correlation from the UALCAN. (A) gene expression correlation between CHST4 and CLIP2 in CCA, (B) gene expression correlation between CHST4 and ELOVL7 in CCA, (C) gene expression correlation between CHST4 and PDGFD in CCA, (D) gene expression correlation between CHST4 and MANEAL in CCA, (E) gene expression correlation between CHST4 and CDKAL1 in CCA, (F) gene expression correlation between CHST4 and SFPQ in CCA, (G) gene expression correlation between CHST4 and GUSBL2 in CCA.

**Fig 4:**
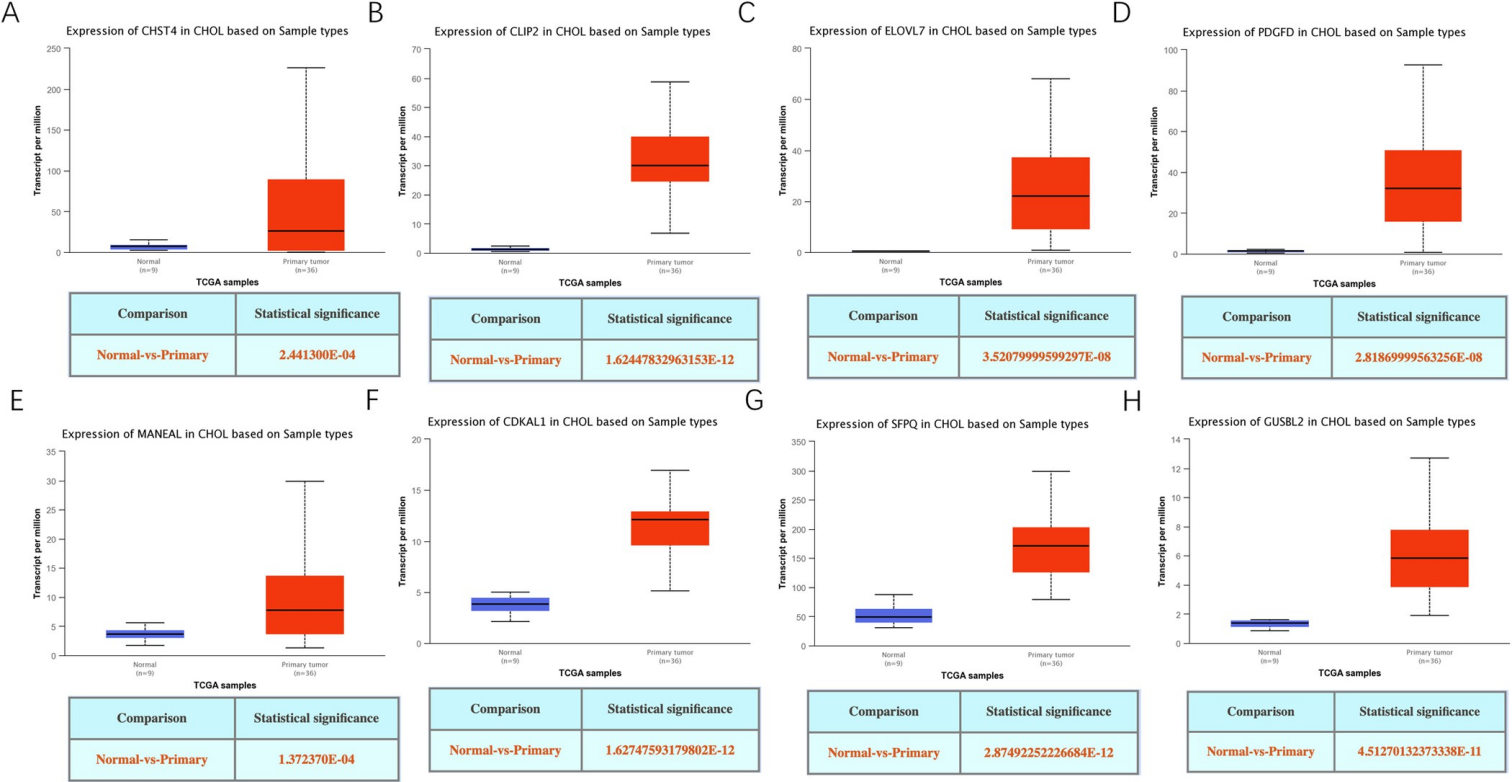
Expression profiles of genes from the UALCAN. (A) expression profiles of CHST4, (B) expression profiles of CLIP2, (C) expression profiles of ELOVL7, (D) expression profiles of PDGFD, (E) expression profiles of MANEAL, (F) expression profiles of CDKAL1, (G) expression profiles of SFPQ, (H) expression profiles of GUSBL2.

Correlated genes of CHST4 were found based on pearson correlation coefficient which was calculated according to gene expression values (TPM) between CHST4 and other genes associated with CCA. Genes with extremely low expression (Median TPM < 0.5) are filtered out. Table 2 showed top 4 genes positively correlated with CHST4 in CCA and top 3 genes negatively correlated with CHST4 in CCA. Expression pattern of correlated genes about CHST4 was delineated (Fig 2). Between the top 4 genes positively correlated and the top 3 genes negatively correlated with CHST4 in CCA, the gene expression correlation are described in Fig 3. The expression profiles of CHST4, CLIP2, ELOVL7, PDGFD, MANEAL, CDKAL1, SFPQ and GUSBL2 were all statistically significant (Fig 4).

**Table 2:** Six correlated genes of CHST4 from the UALCAN.

Genes	Pearson CC
CLIP2	0.64
ELOVL7	0.62
PDGFD	0.6
MANEAL	0.6
CDKAL1	-0.58
SFPQ	-0.54
GUSBL2	-0.54

### PPI network construction and hub genes identification

**Fig 5:**
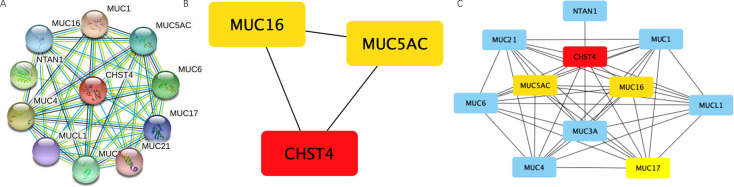
PPI networks of CHST4. (A) all genes in PPI networks, (B) hub genes in PPI networks, (C) hub genes and other genes in PPI networks.

In order to build the protein–protein interaction (PPI) network, we used The Search Tool for the Retrieval of Interacting Networks Genes (STRING). We used cytoscape software 3.8.2. to refine the network (S1 Data). 46 edges and 11 nodes are in the PPI network. We set the average node degree of 8.36 (Fig 5). After that, the rank of connectivity was calculated so finally the top 3 genes were identified (Table 3).

**Table 3:** Top 3 genes in network ranked by degree method.

Rank	Name	Score
1	CHST4	10
2	MUC16	9
2	MUC5AC	9

### Functional enrichment analysis of PPI networks genes

**Fig 6:**
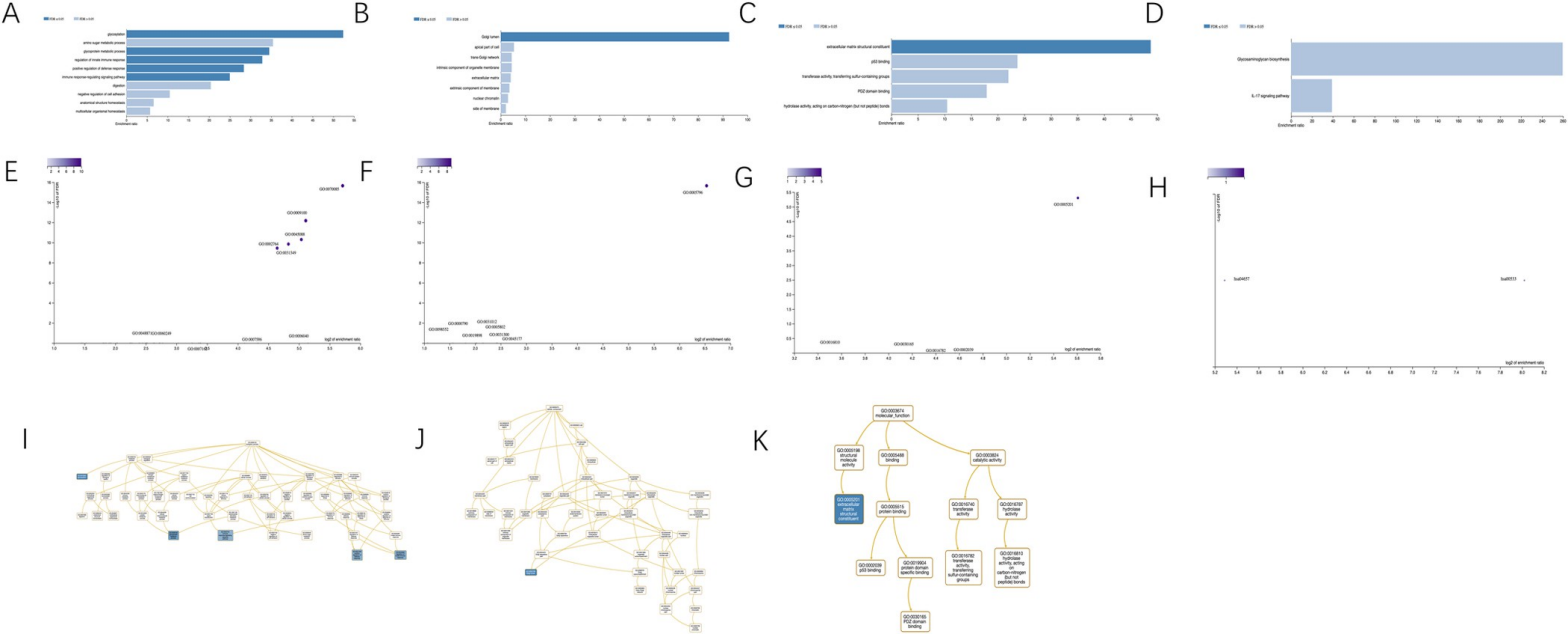
Functional enrichment analysis from the UALCAN. (A) bar chart of BP analysis, (B) bar chart of CC analysis, (C) bar chart of MF analysis, (D) bar chart of KEGG analysis, (E) volcano plot of BP analysis, (F) volcano plot of CC analysis, (G) volcano plot of MF analysis, (H) volcano plot of KEGG analysis, (I) directed acyclic graph of BP analysis, (J) directed acyclic graph of CC analysis, (K) directed acyclic graph of MF analysis.

Functional analyses of ppi networks genes were conducted utilizing the DAVID tools (Table 4). We set gene counts ≥ 4 and genes with FDR < 0.05 as statistically meaningful. From the GO analysis, we found that genes were principally enriched in CCs, which includes golgi lumen, extracellular space and extracellular region. In BP analysis, it suggested that the genes were enriched in o-glycan processing. And in the MF, we found that the in extracellular matrix constituent, lubricant activity genes were enriched. Moreover, the results of KEGG analysis demonstrated that enrichment of genes were not significant. Similar results were obtained using the webgestalt tool for KEGG and GO enrichment analysis (Fig 6).

**Table 4:** Functional analyses of PPI network genes.

Category	Term	Description	Count	FDR
BP term	GO:0016266	O-glycan processing	10	1.67E-20
CC term	GO:0005796	Golgi lumen	9	4.12E-16
CC term	GO:0005615	extracellular space	5	0.030417
CC term	GO:0005576	extracellular region	5	0.043317
MF term	GO:0030197	extracellular matrix constituent, lubricant activity	4	1.54E-08

### CHST4 expression in normal tissues and various tumors

**Fig 7:**
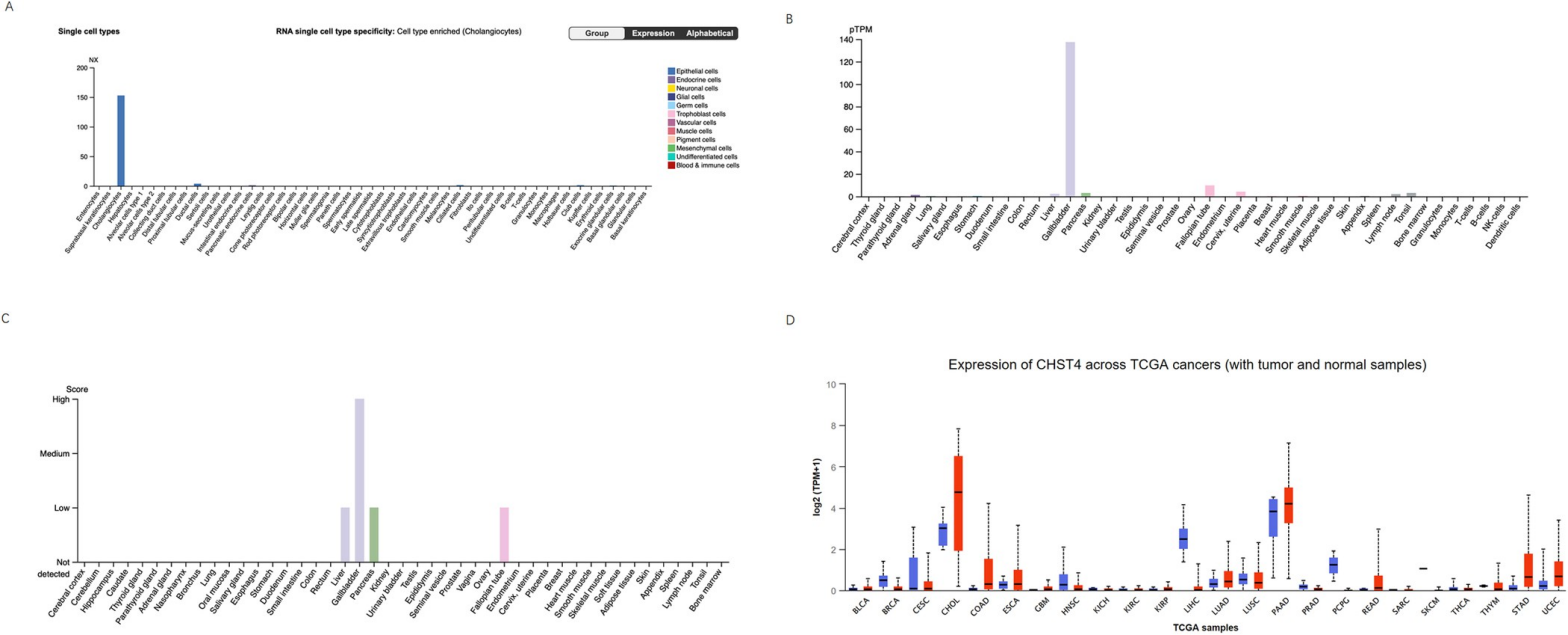
CHST4 expression in normal tissues and various tumors. (A) expression of CHST4 in different normal cells, (B) RNA expression of CHST4 in different normal tissues, (C) protein expression of CHST4 in different normal tissues, (D) expression of CHST4 between tumor and normal samples in TCGA (Red: tumor sample, blue: normal sample).

In normal cells and normal tissues, the expression of CHST4 was observed by HPA (Fig 7). It occurs that CHST4 is very specifically expressed in the bile duct. The expression of CHST4 between tumor and normal samples according to TCGA database are showed in Fig 7 from the UALCAN and the expression of CHST in cholangiocarcinoma was significantly different from that in normal bile ducts.

### Establishment of cholangiocarcinoma animal model

**Fig 8:**
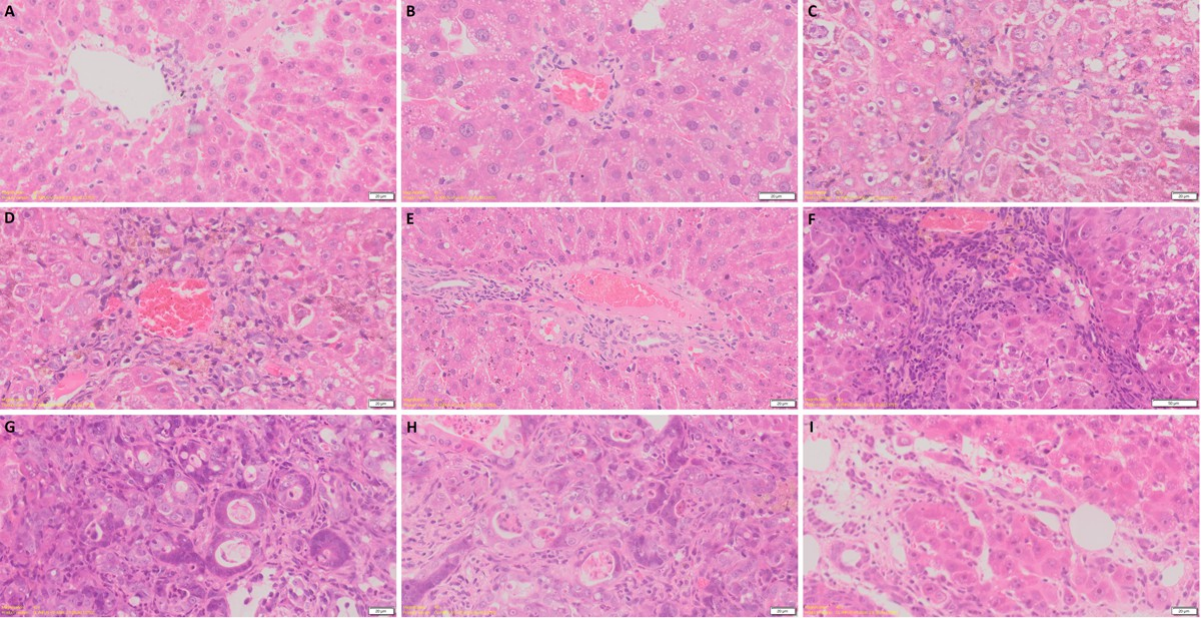
Hematoxylin-eosin staining of CCA model. (A) normal liver of rat, 40x. (B) rat liver after 8 weeks of feeding water containing TAA, 40x. (C) rat liver after 10 weeks of feeding water containing TAA, 40x. (D) rat liver after 12 weeks of feeding water containing TAA, 40x. (E) rat liver after 14 weeks of feeding water containing TAA, 40x. (F) rat liver after 15 weeks of feeding water containing TAA, 40x. (G) rat liver after 16 weeks of feeding water containing TAA, 40x. (H) rat liver after 17 weeks of feeding water containing TAA, 40x. (I) rat liver after 17 weeks of feeding water containing TAA (necrosis), 40x.

**Fig 9:**
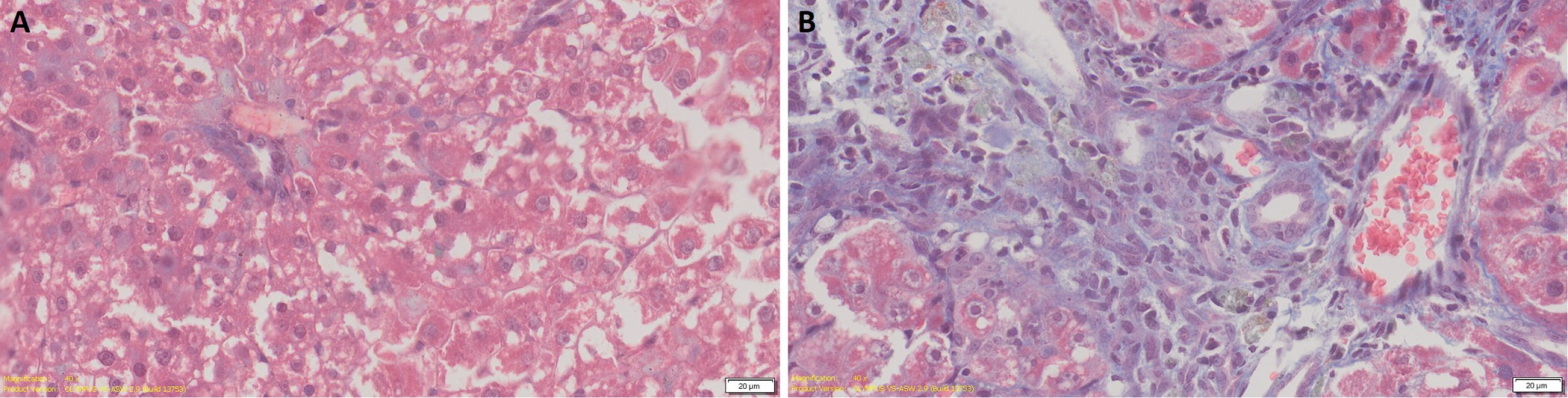
Masson staining of CCA model. (A) rat liver after 5 weeks of feeding water containing TAA, 40x. (B) rat liver after 16 weeks of feeding water containing TAA, 40x.

**Fig 10:**
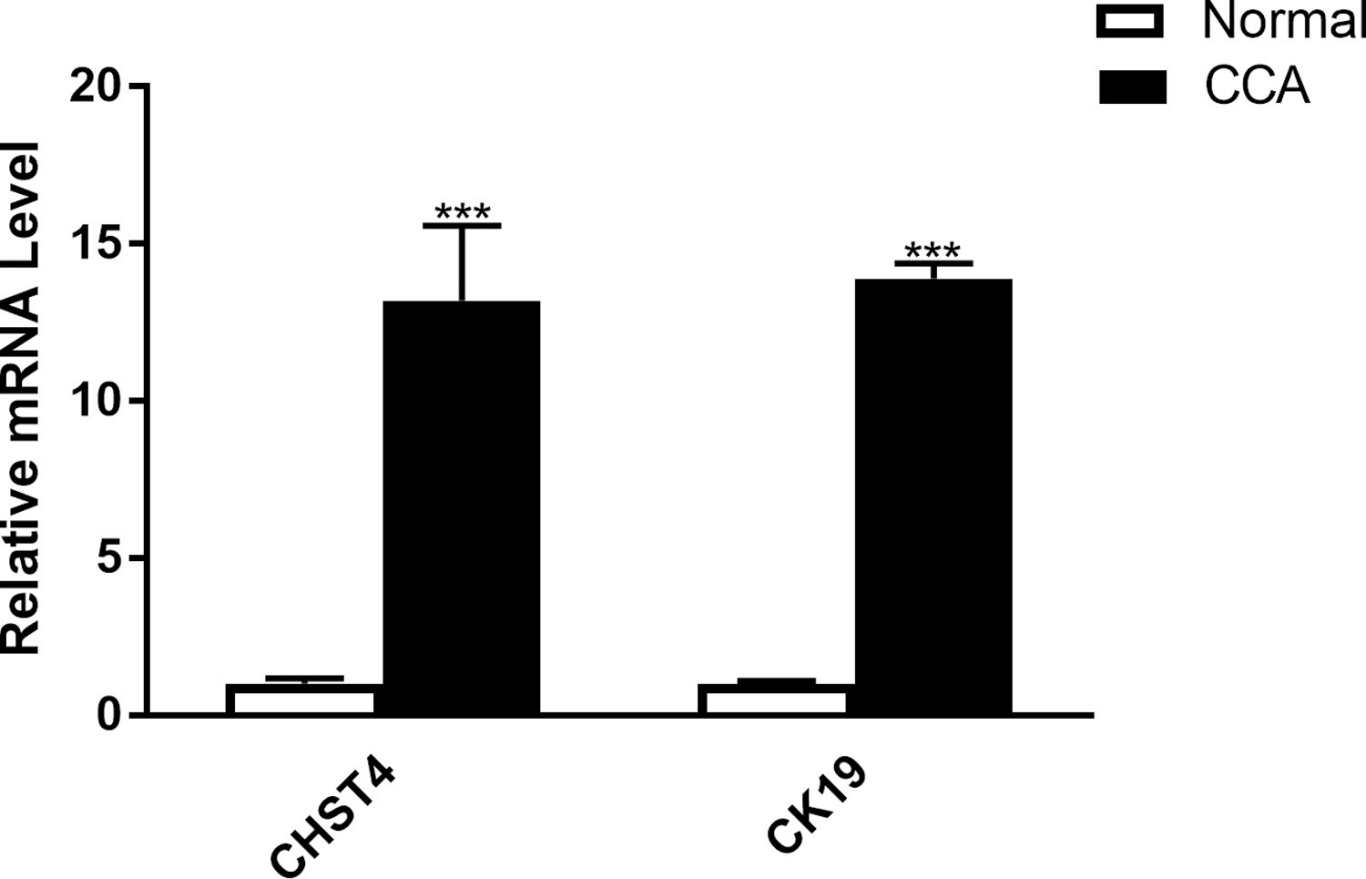
RNA expression of CHST4 and CK19 in normal tissues and CCA. White means normal samples and black means CCA samples (18weeks).

A rat model of cholangiocarcinoma was established by feeding water containing TAA. During the feeding process, we found that the weight difference between the control group and the experimental group became increasingly obvious with the prolongation of the feeding time. In normal liver, few bile ducts can be found around the blood vessels. After 8 weeks of feeding water containing TAA, little bile ducts hyperplasia and fatty degeneration of liver cells was noticed. After 10 weeks, low level of bile duct hyperplasia and liver cells fatty degeneration can be observed. After 12 weeks, bile ducts hyperplasia and fatty degeneration of hepatocytes were obvious. After 14 weeks, there is a large proliferation of bile duct cells. After 15 weeks, the proliferation of bile duct cells is obvious and the deformation of hepatocytes is more obvious. After 16 weeks, fibrosis appeared in the liver and a certain number of rats developed CCA. After 17 weeks of feeding water containing TAA, almost all rats developed tumors and liver cells became necrotic (Fig 8). After 18 weeks of feeding water containing TAA, all rats developed tumors. Fig 9 showed that at the fifth week, there was a small amount of fibroplasia and at the 16th week, a large amount of fibroplasia can be easily observed. The CCA data was referred to Sprague-Dawley (SD) rats fed thioacetamide (TAA) water for 18 weeks. Real-time quantitative polymerase chain reaction showed that not only CHST4 but also CK19 were highly expressed in tumor tissues with statistical significance, verifying the role of CHST4 as the core gene of CCA (Fig 10, S2 Data).

## Discussion

Bile duct cancer, also known as cholangiocarcinoma (CCA), originates from the bile duct cells [18]. CCA is the second largest primary liver tumor after hepatocellular carcinoma and one of the deadliest malignant tumors in humans [19]. CCA has the characteristics of late diagnosis, poor prognosis and low response to existing treatments [20]. At the same time, the incidence rate of CCA has increased significantly, especially in western countries. CCA is a nonpainful but lethal tumor, so at an advanced stage it is usually diagnosed if surgery isn’t available [21]. Surgical resection is the only effective treatment option, which is suitable for only 20% of cases [22]. Patients who are not suitable for surgery receive palliative treatment of gemcitabine combined with cisplatin (GEMCIS). High recurrence rate after tumor resection and chemotherapy resistance lead to an extremely poor prognosis for CCA. Thus, it shows the necessity to figure out novel effective curative targets for CCA treatment [23].

CHST4 (carbohydrate sulfotransferase 4, GlcNAc6ST2), a special type in the CHST protein family, catalyzes the diversion of sulfate. Also, it eventually acts as L-selectin ligand which are present in high endothelial cells (HEV) [24, 25]. So CHST4 makes contribution to lymphocyte homing [26, 27]. Previous reports have already indicated that CHST4 was preferentially expressed in HCC, and it may function as a wonderful diagnosis signal for hepatocellular carcinoma (HCC) patient survival, with higher expression predicting shorter time-to-relapse (TTR) and overall survival (OS). In the HBV-HCC, it was found that the promoter of CHST4 was hyper-methylated [15]. Additionally, following researches showed that CHST4 is highly detected in various solid cancers. It was detected that CHST4 has a highly expression in mucinous adenocarcinomas, in which core 1- and core 2-based-glycans were prolong by CHST4 through adding sulfate, but not in the nonmalignant tissues [28]. In the serum of uterine cervical and corpus cancer, CHST4 provided respectively higher positive rates than cancer antigen 125 (CA125), while it provided lower positive rates for ovarian cancer than CA125 [29]. Therefore, CHST4 is also a good maker for early stage uterine cervical amd corpus cancer. CHST4 also takes part in tumor by implicated in the ectopic expression of MECA-79, which is a newfangled diagnosis signal [30]. The altered the expression degree of CHST4 was discovered from glioma, where its mutation and amplifications are correlated with poor prognosis [31]. Moreover, in the Opisthorchis viverrini ICC, the expression of genes involved in xenobiotic metabolism, which include CHST4, are elevated [16]. Collectively, it suggests that CHST4 is an enzyme associated with different tumors, which also means it can be used as a biomarker for a variety of cancers.

In this study, potential curative targets and prognostic indicators for who were diagnosed with CCA were tried to be identified. We found differentially expressed genes (DEGs) on the Gene Expression Profiling Interactive Analysis (GEPIA) website between tumor and normal tissue. Then, on The Search Tool for the Retrieval of Interacting Networks Genes (STRING) database, the protein-protein interaction (PPI) network was constructed and the top three central genes (CHST4, MUC16 and MUC5AC) of CCA were screened by Cytoscape (3.8.2). MUC16, also called CA125, was prediction gene of surgically excision about CCA [32]. Serum MUC5AC might be potentially used as a surrogate marker in the diagnosis of CCA. Serum concentration of MUC5AC was potential diagnostic markers of CCA [33]. MUC5AC is tightly connected with the happening of cancers, especially hepatocellular carcinoma and cholangiocarcinoma [34].

On WEB-based Gene SeT AnaLysis Toolkit (WebGestalt) and DAVID, we performed Gene Ontology function and Kyoto Encyclopedia of Genes and Genomes pathway enrichment analysis. After that, we built a model of cholangiocarcinoma and tested whether the gene had potential therapeutic value by Quantitative Real-time PCR (qPCR). In conclusion, this study explored the potential therapeutic targets of CCA through bioinformatics techniques and the construction of cholangiocarcinoma models, and the discovered prognostic gene (CHST4) may be used for research and development of new clinical drugs to alleviate the urgent situation of low survival rate in CCA patients.

## Supporting information

S1 Data Protein–protein interaction (PPI) networks data. (TSV)

S2 Data Real-time quantitative polymerase chain reaction data. (XLSX)

## References

[1] Rizvi S, Khan SA, Hallemeier CL, Kelley RK, Gores GJ. Cholangiocarcinoma—evolving concepts and therapeutic strategies. Nat Rev Clin Oncol. 2018;15(2):95–111. doi: 10.1038/nrclinonc.2017.157 28994423 PMC5819599

[2] Banales JM, Marin JJG, Lamarca A, Rodrigues PM, Khan SA, Roberts LR, et al. Cholangiocarcinoma 2020: the next horizon in mechanisms and management. Nat Rev Gastroenterol Hepatol. 2020;17(9):557–88. doi: 10.1038/s41575-020-0310-z 32606456 PMC7447603

[3] Louis C, Papoutsoglou P, Coulouarn C. Molecular classification of cholangiocarcinoma. Curr Opin Gastroenterol. 2020;36(2):57–62. doi: 10.1097/MOG.0000000000000611 31895230

[4] Saha SK, Zhu AX, Fuchs CS, Brooks GA. Forty-Year Trends in Cholangiocarcinoma Incidence in the U.S.: Intrahepatic Disease on the Rise. Oncologist. 2016;21(5):594–9. doi: 10.1634/theoncologist.2015-0446 27000463 PMC4861366

[5] Yang JD, Kim B, Sanderson SO, Sauver JS, Yawn BP, Larson JJ, et al. Biliary tract cancers in Olmsted County, Minnesota, 1976–2008. Am J Gastroenterol. 2012;107(8):1256–62. doi: 10.1038/ajg.2012.173 22751468 PMC3654834

[6] Chaiteerakij R, Yang JD, Harmsen WS, Slettedahl SW, Mettler TA, Fredericksen ZS, et al. Risk factors for intrahepatic cholangiocarcinoma: association between metformin use and reduced cancer risk. Hepatology. 2013;57(2):648–55. doi: 10.1002/hep.26092 23055147 PMC3565026

[7] Khan SA, Tavolari S, Brandi G. Cholangiocarcinoma: Epidemiology and risk factors. Liver Int. 2019;39 Suppl 1:19–31. doi: 10.1111/liv.14095 30851228

[8] Bertuccio P, Malvezzi M, Carioli G, Hashim D, Boffetta P, El-Serag HB, et al. Reply to: "Global trends in mortality from intrahepatic and extrahepatic cholangiocarcinoma". J Hepatol. 2019;71(6):1262–3. doi: 10.1016/j.jhep.2019.08.033 31564445

[9] Valle J, Wasan H, Palmer DH, Cunningham D, Anthoney A, Maraveyas A, et al. Cisplatin plus gemcitabine versus gemcitabine for biliary tract cancer. N Engl J Med. 2010;362(14):1273–81. doi: 10.1056/NEJMoa0908721 20375404

[10] Luo X, Campbell NA, He L, O’Brien DR, Singer MS, Lemjabbar-Alaoui H, et al. Sulfatase 2 (SULF2) Monoclonal Antibody 5D5 Suppresses Human Cholangiocarcinoma Xenograft Growth Through Regulation of a SULF2-Platelet-Derived Growth Factor Receptor Beta-Yes-Associated Protein Signaling Axis. Hepatology. 2021;74(3):1411–28. doi: 10.1002/hep.31817 33735525 PMC9075007

[11] Bekaii-Saab TS, Bridgewater J, Normanno N. Practical considerations in screening for genetic alterations in cholangiocarcinoma. Ann Oncol. 2021;32(9):1111–26. doi: 10.1016/j.annonc.2021.04.012 33932504

[12] Long J, Huang S, Bai Y, Mao J, Wang A, Lin Y, et al. Transcriptional landscape of cholangiocarcinoma revealed by weighted gene coexpression network analysis. Brief Bioinform. 2021;22(4). doi: 10.1093/bib/bbaa224 33051665

[13] Uhlén M, Björling E, Agaton C, Szigyarto CA, Amini B, Andersen E, et al. A human protein atlas for normal and cancer tissues based on antibody proteomics. Mol Cell Proteomics. 2005;4(12):1920–32. doi: 10.1074/mcp.M500279-MCP200 16127175

[14] Veerman K, Tardiveau C, Martins F, Coudert J, Girard JP. Single-Cell Analysis Reveals Heterogeneity of High Endothelial Venules and Different Regulation of Genes Controlling Lymphocyte Entry to Lymph Nodes. Cell Rep. 2019;26(11):3116–31.e5. doi: 10.1016/j.celrep.2019.02.042 30865898

[15] Zhang L, Fan Y, Wang X, Yang M, Wu X, Huang W, et al. Carbohydrate Sulfotransferase 4 Inhibits the Progression of Hepatitis B Virus-Related Hepatocellular Carcinoma and Is a Potential Prognostic Marker in Several Tumors. Front Oncol. 2020;10:554331. doi: 10.3389/fonc.2020.554331 33178582 PMC7593664

[16] Jinawath N, Chamgramol Y, Furukawa Y, Obama K, Tsunoda T, Sripa B, et al. Comparison of gene expression profiles between Opisthorchis viverrini and non-Opisthorchis viverrini associated human intrahepatic cholangiocarcinoma. Hepatology. 2006;44(4):1025–38. doi: 10.1002/hep.21330 17006947

[17] Yeh CN, Maitra A, Lee KF, Jan YY, Chen MF. Thioacetamide-induced intestinal-type cholangiocarcinoma in rat: an animal model recapitulating the multi-stage progression of human cholangiocarcinoma. Carcinogenesis. 2004;25(4):631–6. doi: 10.1093/carcin/bgh037 14656942

[18] Pant K, Peixoto E, Richard S, Biswas A, O’Sullivan MG, Giama N, et al. Histone Deacetylase Sirtuin 1 Promotes Loss of Primary Cilia in Cholangiocarcinoma. Hepatology. 2021;74(6):3235–48. doi: 10.1002/hep.32080 34322899

[19] Banales JM, Cardinale V, Carpino G, Marzioni M, Andersen JB, Invernizzi P, et al. Expert consensus document: Cholangiocarcinoma: current knowledge and future perspectives consensus statement from the European Network for the Study of Cholangiocarcinoma (ENS-CCA). Nat Rev Gastroenterol Hepatol. 2016;13(5):261–80. doi: 10.1038/nrgastro.2016.51 27095655

[20] Blechacz B. Cholangiocarcinoma: Current Knowledge and New Developments. Gut Liver. 2017;11(1):13–26. doi: 10.5009/gnl15568 27928095 PMC5221857

[21] Lobe C, Vallette M, Arbelaiz A, Gonzalez-Sanchez E, Izquierdo L, Pellat A, et al. Zinc Finger E-Box Binding Homeobox 1 Promotes Cholangiocarcinoma Progression Through Tumor Dedifferentiation and Tumor-Stroma Paracrine Signaling. Hepatology. 2021;74(6):3194–212. doi: 10.1002/hep.32069 34297412

[22] Lee YT, Wang JJ, Luu M, Noureddin M, Nissen NN, Patel TC, et al. Comparison of Clinical Features and Outcomes Between Intrahepatic Cholangiocarcinoma and Hepatocellular Carcinoma in the United States. Hepatology. 2021;74(5):2622–32. doi: 10.1002/hep.32007 34114675

[23] Gentilini A, Lori G, Caligiuri A, Raggi C, Di Maira G, Pastore M, et al. Extracellular Signal-Regulated Kinase 5 Regulates the Malignant Phenotype of Cholangiocarcinoma Cells. Hepatology. 2021;74(4):2007–20. doi: 10.1002/hep.31888 33959996 PMC8518067

[24] Uchimura K, Gauguet JM, Singer MS, Tsay D, Kannagi R, Muramatsu T, et al. A major class of L-selectin ligands is eliminated in mice deficient in two sulfotransferases expressed in high endothelial venules. Nat Immunol. 2005;6(11):1105–13. doi: 10.1038/ni1258 16227986

[25] Kawashima H, Petryniak B, Hiraoka N, Mitoma J, Huckaby V, Nakayama J, et al. N-acetylglucosamine-6-O-sulfotransferases 1 and 2 cooperatively control lymphocyte homing through L-selectin ligand biosynthesis in high endothelial venules. Nat Immunol. 2005;6(11):1096–104. doi: 10.1038/ni1259 16227985

[26] Tsuboi K, Hirakawa J, Seki E, Imai Y, Yamaguchi Y, Fukuda M, et al. Role of high endothelial venule-expressed heparan sulfate in chemokine presentation and lymphocyte homing. J Immunol. 2013;191(1):448–55. doi: 10.4049/jimmunol.1203061 23733868 PMC3694755

[27] Ohmichi Y, Hirakawa J, Imai Y, Fukuda M, Kawashima H. Essential role of peripheral node addressin in lymphocyte homing to nasal-associated lymphoid tissues and allergic immune responses. J Exp Med. 2011;208(5):1015–25. doi: 10.1084/jem.20101786 21518796 PMC3092357

[28] Yu SY, Hsiao CT, Izawa M, Yusa A, Ishida H, Nakamura S, et al. Distinct substrate specificities of human GlcNAc-6-sulfotransferases revealed by mass spectrometry-based sulfoglycomic analysis. J Biol Chem. 2018;293(39):15163–77. doi: 10.1074/jbc.RA118.001937 30093410 PMC6166739

[29] Seko A, Kataoka F, Aoki D, Sakamoto M, Nakamura T, Hatae M, et al. N-Acetylglucosamine 6-O-sulfotransferase-2 as a tumor marker for uterine cervical and corpus cancer. Glycoconj J. 2009;26(8):1065–73. doi: 10.1007/s10719-008-9227-4 19156517

[30] Okayama H, Kumamoto K, Saitou K, Hayase S, Kofunato Y, Sato Y, et al. Ectopic expression of MECA-79 as a novel prognostic indicator in gastric cancer. Cancer Sci. 2011;102(5):1088–94. doi: 10.1111/j.1349-7006.2011.01895.x 21281400 PMC11159981

[31] Subbarayan K, Seliger B. Tumor-dependent Effects of Proteoglycans and Various Glycosaminoglycan Synthesizing Enzymes and Sulfotransferases on Patients’ Outcome. Curr Cancer Drug Targets. 2019;19(3):210–21. doi: 10.2174/1568009618666180706165845 29984655

[32] Fang T, Wang H, Wang Y, Lin X, Cui Y, Wang Z. Clinical Significance of Preoperative Serum CEA, CA125, and CA19-9 Levels in Predicting the Resectability of Cholangiocarcinoma. Dis Markers. 2019;2019:6016931. doi: 10.1155/2019/6016931 30863466 PMC6378785

[33] Xuan J, Li J, Zhou Z, Zhou R, Xu H, Wen W. The diagnostic performance of serum MUC5AC for cholangiocarcinoma: A systematic review and meta-analysis. Medicine (Baltimore). 2016;95(24):e3513. doi: 10.1097/MD.0000000000003513 27310944 PMC4998430

[34] Kasprzak A, Adamek A. Mucins: the Old, the New and the Promising Factors in Hepatobiliary Carcinogenesis. Int J Mol Sci. 2019;20(6). doi: 10.3390/ijms20061288 30875782 PMC6471604

